# Healthcare system responsiveness in Jiangsu Province, China

**DOI:** 10.1186/s12913-017-1980-2

**Published:** 2017-01-13

**Authors:** Jianqian Chao, Boyang Lu, Hua Zhang, Liguo Zhu, Hui Jin, Pei Liu

**Affiliations:** 1 Department of Medical insurance, Key Laboratory of Environmental Medicine Engineering of Ministry of Education, School of Public Health, Southeast University, Nanjing, Jiangsu China; 2 Jiangsu Provincial Center for Disease Control and Prevention, Nanjing, Jiangsu China; 3 Department of Epidemiology and Biostatistics, School of Public Health, Southeast University, Nanjing, Jiangsu China; 4 Xuzhou Medical University, Xuzhou, Jiangsu China

**Keywords:** Health system performance, Responsiveness, People characteristics, Canonical correlation analysis

## Abstract

**Background:**

The perceived responsiveness of a healthcare system reflects its ability to satisfy reasonable expectations of the public with respect to non-medical services. Recently, there has been increasing attention paid to responsiveness in evaluating the performance of a healthcare system in a variety of service settings. However, the factors that affect the responsiveness have been inconclusive so far and measures of improved responsiveness have not always thoroughly considered the factors. The aim of this study was to evaluate both the responsiveness of the healthcare system in Jiangsu Province, China, the factors that influence responsiveness and the measures of improved responsiveness considering it, as determined by a responsiveness survey.

**Methods:**

A multistage, stratified random sampling method was used to select 1938 adult residents of Jiangsu Province in 2011. Face-to-face interviews were conducted using a self-designed questionnaire modeled on the World Health Organization proposal. The final analysis was based on 1783 (92%) valid questionnaires. Canonical correlation analysis was used to assess the factors that affect responsiveness.

**Results:**

The average score of all responsiveness-related domains in the surveyed healthcare system was satisfactory (7.50 out of a maximum 10.0). The two highest scoring domains were dignity and confidentiality, and the two lowest scoring domains choice and prompt attention. The factors affecting responsiveness were age, regional economic development level, and geographic area (urban vs. rural). The responsiveness regarding basic amenities was rated worse by the elderly than by younger respondents. Responsiveness ranked better by those with a poorer economic status. Choice in cities was better than in rural regions.

**Conclusions:**

The responsiveness of the Jiangsu healthcare system was considered to be satisfactory but could be improved by offering greater choice and providing more prompt attention. Perceptions of healthcare system responsiveness differ with age, regional economic development level, and geographic area (urban vs. rural). Measures to increase the perceived level of responsiveness include better service at higher level hospitals, shorter waiting time, more hospitals in rural regions, an improved medical environment, and provision of infrastructures that makes the medical environments more comfortable.

**Electronic supplementary material:**

The online version of this article (doi:10.1186/s12913-017-1980-2) contains supplementary material, which is available to authorized users.

## Background

The three intrinsic goals of a healthcare system are good health, responsiveness, and financial fairness, as suggested in the World Health Report 2000 [1]. Responsiveness is defined as meeting the legitimate expectations of patients and their families regarding non-medical aspects of the healthcare system [2]. Of these three goals, responsiveness can be most efficiently manipulated to improve a healthcare system. With consumers’ growing awareness of their rights and increasing competition among healthcare organizations, patients’ views are increasingly being taken into account in healthcare policy making. Accordingly, responsiveness has become a major consideration in evaluating the quality of a healthcare system. Indeed, one study showed that when responsiveness was improved, other healthcare goals were enhanced as well [3].

Responsiveness has been evaluated in several studies [4–9], some of which have shown that the levels of healthcare system responsiveness differed between countries [7—9]. The World Health Organization (WHO) estimated responsiveness and healthcare distribution in 191 countries and described its strategy for improving the measurement of responsiveness in future evaluations [10]. Coulter et al. conducted a telephone survey in Europe based on a random sample of 8119 people aged 16 years and over in 2005. They found that many European patients wanted a more autonomous role in healthcare decision making and that policy makers and clinicians should consider measures aimed at narrowing the gap between public expectations and the patient experience [11]. However, the factors affecting healthcare system responsiveness have yet to be determined. The few studies that have considered these factors have mainly focused on the apparent positive association between healthcare spending per capita and responsiveness, or on insured users’ perception of the responsiveness of their healthcare services [1, 12, 13]. Robone et al. investigated the potential country-level drivers of healthcare system responsiveness in 2011 and suggested that improvements in responsiveness may require higher spending levels. Expansion of the non-public provision of healthcare, in which there is increased patient choice, may also serve to improve responsiveness. However, these inferences are tentative and require further study [14]. Jung et al. showed that patient characteristics were an important determinant of preferences in many aspects of primary healthcare [15]. However, later studies reported weak associations between the relative importance given to non-medical quality dimensions and the individual characteristics of the patient [16]. In fact, in many developing countries the responsiveness of the healthcare system was ignored and was inadequate to meet patients’ non-medical expectations [17].

Responsiveness has also been studied in China [18–21], where healthcare system reform has included measures aimed at improving the healthcare system. Jiang investigated the level of responsiveness in Shanghai [19], whereas Lu et al. and Wang et al. studied the level of responsiveness and factors that affect responsiveness in Anhui Province and the city of Zhengzhou respectively [18, 21]. Nonetheless, the factors that affect the responsiveness have been inconclusive so far and measures of improved responsiveness have not always thoroughly considered the factors. Thus, in this study we used a multistage, stratified random sampling method to evaluate responsiveness levels in Jiangsu Province of China, to identify the factors that determine responsiveness. We expect that our results will contribute to a better interpretation of healthcare needs and to defining the measures needed to improve responsiveness, which in turn improve healthcare system performance.

## Methods

### Sample and procedure

This study was conducted in three prefectures (cities) in Jiangsu Province, China, during July 2011. Jiangsu is located in southeastern China and is one of the most developed provinces in the country. Its healthcare system consists of large hospitals as well as basic medical and health institutions, including community healthcare facilities, town hospitals, public health institutions, and other medical facilities.

A multistage, stratified random sample of the general population was carried out in the three prefectures of Jiangsu, which differed in their levels of regional economic development. In each prefecture, one administrative village from a rural area and one community from an urban area were randomly selected. According to Hatcher, the sample size should be 5–10 times the variable number [22]. In this study, the variable number was 75 with 8 × 75 = 600 respondents in each prefecture (city). Allowing for a 10% increase, the sample size was 1980 participants. Within each city, 660 residents 18 years of age or older were randomly selected using a random number system. Care was taken to obtain a representative age and sex distribution within the target population. First, one village and one community were randomly selected as described above. We then generated a list of numbers representing the different residents through coding. The list was generated using a random numbers table that considered the age and sex of the residents. Participants were then selected according to the random numbers. Temporary residents were excluded. Of the 1980 residents initially selected, 1938 participated in this study (97.9% response rate), which was based on a self-designed questionnaire (see below). Of the submitted questionnaires, 1783 were valid (validity rate 92.0%). In addition, trained investigators conducted personal interviews with participants informing to the village or community office.

### Outcome measures

The self-designed questionnaire was based on the healthcare system household questionnaire of the WHO [1], which had been modified for our study purpose. It queried perceptions of responsiveness in terms of the characteristics of respondents, including their age, sex, educational level, marital status, family income, occupation, and type of medical insurance. using seven domains: dignity, confidentiality, communication, autonomy, choice of care provider, prompt attention, and quality of basic amenities.

Respondents were asked to rate the following: 1) being greeted and spoken to respectfully; 2) respect for intimacy during physical examination and care; 3) confidentiality of personal information; 4) the possibility of obtaining information about other types of treatment or tests; 5) participation in decision making regarding healthcare or treatment; 6) the clarity of explanations given by healthcare providers; 7) sufficient time to ask questions about the health problem or its treatment; 8) the ability to choose the healthcare provider; 9) waiting time before being attended; 10) cleanliness of the facility, including restrooms; and 11) available space in waiting and examination rooms. The questionnaire consisted of 15 items, divided into the seven domains mentioned above, as follows: dignity (2 questions), confidentiality (1 question), communication (3 questions), autonomy (1 question), choice (2 questions), prompt attention (4 questions), and quality of basic amenities (2 questions). We used a scale ranging from Never = 0, Sometimes = 5, Usually = 7.5, and Always = 10 Additional file 1. Each domain was rated using a 10-point scoring system in which Best = 10 and Worst = 0; scores of 7.5 and above were considered satisfactory, less than 7.5 to 5 moderately satisfactory, and less than 5 unsatisfactory. We calculated each domain score using averages. The weighted mean score of each responsiveness domain was then determined, as recommended by the WHO (Valentine, Silva, Murray, 2000). The higher the score, the better the responsiveness [23].

Before the investigation, a pilot study was conducted on a small sample size. Based on the results, the questionnaire was further improved. At this stage, experts in the field were invited to discuss the design of the questionnaire, including, for example, the need for elimination of inappropriate items. This process ensured the content validity of the questionnaire.

### Data analysis

All analyses were performed using SPSS version 17.0 (SPSS Inc., Chicago, IL, USA) and SAS version 8.1 (SAS Institute Inc., Cary, NC, USA) statistical software. A *P* value ≤  0.05 was considered to indicate statistical significance.

Categorical variables were analyzed using a *χ*2 test and measurement data using a *t*-test. Canonical correlation analysis was used to explore the correlation between respondent characteristics (X set) and responsiveness (Y set), to identify the main factors influencing healthcare system responsiveness.

X = {sex, age, education, family income, medical insurance, regional economic development level, geographic area (urban or rural)} = {X1, *X*2, X3, X4, X5, X6, X7}

Y = {dignity, confidentiality, communication, autonomy, choice, prompt attention, quality of basic amenities} = {Y_1_, Y_2_, Y_3_, Y_4_, Y_5_, Y_6_, Y_7_}

Thus, in the canonical correlation analysis, the canonical linear combination was V_i_ = a_1_X_1_ + a_2_X_2_ + a_3_X_3_ + a_4_X_4_+ a_5_X_5_+ a_6_X_6_+ a_7_X_7_; W_i_ = b_1_Y_1_ + b_2_Y_2_ + b_3_Y_3_ + b_4_Y_4_ + b_5_Y_5_ + b_6_Y_6_ + b_7_Y_7_ (a, b: the standardized canonical correlation coefficient, i = 1, 2,…; V_i_, W_i_ canonical correlation coefficient).

## Results

### Participant characteristics

Characteristics of the 1783 respondents who provided valid questionnaires are listed in Table 1. Within this group, the average age was 39.2 years (SD = 13.4); 59.6% were female; 71.6% were married; 77.1% had completed high school or junior college; 71.5% had an average family economic status. The two main types (67.8%) of medical insurance among respondents were urban employee insurance and the recently introduced rural corporate medical insurance.

**Table 1:** Participant characteristics

	Number (*n* = 1783)	Percentage (%)
Sex		
Male	881	49.4
Female	902	59.6
Age (years)		
Mean ± SD	39.2 ± 13.4	
range	18–78	
Marriage		
Married	1276	71.6
Unmarried	480	26.9
Divorced or widowed	27	1.5
Education		
Elementary school and under	203	11.4
Middle school and College	1375	77.1
University and higher	205	11.5
Occupation		
Worker	556	31.2
Farmer	170	9.5
Civil service	188	10.5
Technical staff	162	9.1
Service personnel	216	12.1
Retiree	69	3.9
Unemployed	53	3.0
Students and children	279	15.6
Other	90	5.1
Family income		
Very poor	49	2.8
Below average	283	15.9
Average	1275	71.5
Good	153	8.6
Very good	22	1.2
Medical insurance		
Free medical care	53	3.0
Basic medical insurance for urban residents	329	18.5
Basic medical insurance for urban employee	624	35.0
New rural cooperative medical insurance	584	32.7
Self-pay and other	193	10.8

### Scores of the seven domains of healthcare system responsiveness

Table 2 presents results of the evaluation of healthcare system responsiveness with respect to the seven domains mentioned above. Dignity had the highest score as an indicator of responsiveness (8.44 out of a maximum 10.0) and was ranked as “satisfactory” by 74.8% of respondents. This was followed by confidentiality and communication, each with a score of 8.0 and ranked as “satisfactory” by 72.1% and 66.5% of respondents, respectively. Choice had the lowest score (6.88) and was considered “satisfactory” only by 52.5% of respondents; prompt attention had a similar score (6.89) and was ranked as “satisfactory” by 54.2%. The difference between scores of the seven domains and responsiveness as a whole was significant (*P*  <  0.001). The overall average score of healthcare system responsiveness in Jiangsu Province was 7.50.

**Table 2:** Response frequency and scores of healthcare system indicators

Domain	Satisfactory		Moderate		Unsatisfactory		Score	Rank by score
	n	%	n	%	n	%		
dignity	1333	74.8	345	19.3	105	5.9	8.44	1
confidentiality	1286	72.1	280	15.7	217	12.2	8.00	2
autonomy	1107	62.1	469	26.3	207	11.6	7.52	4
communication	1185	66.5	485	27.2	113	6.3	8.00	2
choice	937	52.5	579	32.5	267	15.0	6.88	7
prompt attention	966	54.2	527	29.5	290	16.3	6.89	6
basic amenities	901	50.5	781	43.8	101	5.7	7.25	5

The formula used to score healthcare system responsiveness was as follows [10]:

$$ \begin{array}{l}\mathrm{Y}=0.125*{\mathrm{V}}_1+0.125*{\mathrm{V}}_2+0.125*{\mathrm{V}}_3+0.125*{\mathrm{V}}_4+0.25*{\mathrm{V}}_5+0.15*{\mathrm{V}}_6+0.10*{\mathrm{V}}_7\\ {}=0.125*8.44+0.125*8.0+0.125*7.52+0.125*8.0+0.25*6.89+0.15*7.25+0.10*6.88=7.50\end{array} $$

weight determined on the basis of the WHO questionnaire and modified based on expert opinions.

### Factors affecting responsiveness

Canonical correlation analysis was used to identify the effect of participant characteristics (X set) on responsiveness (Y set). The results showed that among the seven pairs of canonical variables, the first three (r1, r2, r3) differed significantly (*P*  <  0.01). The canonical correlation coefficients of the three pairs were 0.338, 0.179, and 0.144, which explained 66.6%, 17.0%, and 10.9% of the variance, respectively. The cumulative contribution rate was 94.5% (Table 3).

**Table 3:** Canonical correlation analysis between participant characteristics variables and healthcare system responsiveness

Serial number	Canonical coefficient	Contribution rate	Cumulative contribution rate	*F*	*r*	*P* value
1	0.338	0.666	0.666	6.555	49	0.000
2	0.179	0.170	0.836	3.044	36	0.000
3	0.144	0.109	0.945	2.152	25	0.001
4	0.085	0.038	0.983	1.132	16	0.318
5	0.042	0.009	0.992	0.624	9	0.778
6	0.034	0.006	0.998	0.641	4	0.634
7	0.019	0.002	1.000	0.620	1	0.431

In this study, the demographic characteristics and response dimensions consisted of several variables such that neither a simple correlation nor multiple linear regression analysis was appropriate. Instead, we used canonical correlation analysis to reflect the relationship between demographic characteristics and the responses. Standardized canonical correlation coefficients of the canonical variables are presented in Table 4. The first canonical variable of participant characteristics, V1, was mainly represented by the level of regional economic development; V2 was represented by urban versus rural locations, and V3 by age. The first canonical variable in healthcare system responsiveness, W1, was mainly represented by prompt attention whereas W2 was represented by choice and W3 by basic amenities. The first canonical correlation indicated a positive correlation between regional economic development level and prompt attention, with responsiveness ranked better by those with a poorer economic status. The second canonical correlation showed greater choice in urban areas, and the third showed that responsiveness in terms of basic amenities was worse for the elderly.

**Table 4:** Standardized canonical correlation coefficients of participant characteristics variables and healthcare system responsiveness

Participant characteristics variables	V1	V2	V3	Healthcare system responsiveness variables	W1	W2	W3
Sex	−0.056	−0.022	0.450	Dignity	0.041	0.429	0.148
Age	0.359	0.313	0.547	Autonomy	−0.492	−0.164	0.088
Education	−0.385	0.168	−0.259	Confidentiality	−0.038	−0.642	−0.371
Family income	0.261	0.319	0.177	Communication	0.075	0.184	−0.707
Medical insurance	0.049	0.138	0.133	Choice	0.254	−0.787	−0.026
Geographic location (urban vs. rural)	0.310	0.671	−0.509	Prompt attention	0.822	−0.137	0.013
regional economic development level	0.581	−0.586	−0.294	Basic amenities	−0.136	0.063	0.990

## Discussion

Responsiveness with respect to a healthcare system is defined as the extent to which that system satisfies the population’s common, reasonable expectations for non-medical aspects of care. In China, the healthcare system has undergone recent reform, including measures to accelerate construction of a basic medical security system, establishment of a National Essential Medicines Policy, improvements in basic medical and healthcare services, and gradual equalization of basic public health services. To identify problems affecting the Chinese healthcare system and those actions that will lead to its improvement, we used a multistage, stratified random sampling method to evaluate the system’s level of responsiveness. Specifically, we assessed the factors that affect responsiveness in a survey of a random sample of the population in Jiangsu Province, China.

The responsiveness of the healthcare system in Jiangsu achieved a score of 7.50, which indicated a satisfactory level. In the evaluation of seven domains contributing to healthcare system responsiveness, dignity and confidentiality were ranked highest and choice and prompt attention lowest. Ratings of healthcare system responsiveness differ across different studies [5, 19, 24–26]. The relative rankings in our study were the same as those reported for Israel [24]. In Anhui Province of China, dignity ranked highest and choice lowest [18]. In South Africa, freedom to choose the healthcare provider was rated as of least importance whereas in our study, dignity and confidentiality were of greatest importance [27]. By contrast, in the results for 35 countries reported by the WHO, social support and confidentiality received the highest scores and autonomy and basic amenities the lowest [25]. In 16 OECD countries, the two highest scoring factors were choice and dignity, and the two lowest scoring factors were prompt attention and communication [26]. Our results also differed from the relative rankings reported from a 2003 survey in Shanghai, in which basic amenities and autonomy scored lowest, social support and dignity highest [19]. In relative rankings reported from a 2000 survey in Shandong Province, autonomy and dignity scored lowest, social support and confidentiality highest [3]. In our study population, patients were assigned healthcare providers and thus had very little free choice. An additional issue was long waiting time before being attended to in the hospital. Both of these factors were also identified as problems in the Shanghai study [19]. Our study participants were relatively healthy such that their healthcare needs could be met by the existing medical institutions in their respective prefectures; only rarely was care required from another hospital or medical institution. Nonetheless, our results revealed that two important determinants of responsiveness, choice and prompt attention, required improvement in the three prefectures of Jiangsu Province surveyed.

Our study used the canonical correlation method to analyze those factors that affect healthcare system responsiveness. Recognition of an association between the characteristics of the population and the preference for healthcare system services will aid healthcare officials to determine needs accordingly and to set priorities. Some factors considered in this study, such as age, sex, and education, have previously been shown to be associated with healthcare system preferences in other countries [9, 13, 28]; however, in 16 OECD countries surveyed, there were few consistent patterns [26]. Our results showed that those factors that defined responsiveness differed significantly depending on the characteristics of respondents. Among those who were poorer economic status, prompt attention rated higher. This may reflect the fact that patients in the areas of poorer economic status often present to a community healthcare center or to a county or village hospital, whereas patients in the areas of better economic status will typically visit a higher level hospital where the large number of patients and complex admission process lead to the “three longs and a short” phenomenon: long registration time, long waiting time to see a doctor, long time waiting to be billed, and a short time spent seeing the doctor. It also might be that patients from better economic status areas have higher demands. Rice et al. reported that individuals with higher incomes are more likely to report very good responsiveness and less likely to report moderate responsiveness than individuals with lower incomes, according to a survey across 54 countries from the World Health Survey (WHS) [9]. We found that choice in urban regions was better than that in rural regions, which reflected the fact that there are fewer hospitals in the latter areas. In cities, there are more facilities and personnel and better local access, which results in more choice. Age was positively associated with basic amenities, suggesting that older people have higher expectations about the medical environment. This is consistent with the conclusion by Coulter that the gap between patients’ expectations and the realities of care is one of the factors that strongly influences perceptions of responsiveness [11]. Our results also indicated that a comfortable medical environment requires improvements in the related infrastructure.

Our study had several limitations. First, the responsiveness survey did not distinguish between hospitals, primary healthcare facilities, medical centers, and other institutions that provide healthcare services [29]. However, these facilities differ in their responsiveness. Researchers have attempted to account for these differences in studies that have yielded different results [30, 31]. Second, responsiveness evaluations emphasize common and reasonable expectations; however, these are influenced by the characteristics of respondents, such as their age, education level, and income, thus introducing bias in expectations [19, 32]. Also, in our study, we investigated responsiveness of the general population rather than that of patients attending a specific hospital, public health service, or health center, which may have produced different results. Third, our sample size was relatively small, and the survey was limited to Jiangsu Province. As noted above, our results had similarities to, but also differences from, the findings reported for other provinces and cities of China. Finally, a standardized definition of responsiveness and a standardized approach to its measurement are needed, including the ability to account for the healthcare expectations of survey participants [33].

## Conclusions

We studied the responsiveness of adult residents (18 years of age and older) in three prefectures of Jiangsu Province. Our results showed that the responsiveness of the Jiangsu healthcare system was considered to be satisfactory but could be improved by offering greater choice and providing more prompt attention. Perceptions of healthcare system responsiveness differed with age, regional economic development level, and geographic area (urban vs. rural). Healthcare policy makers should take these factors into account when instituting measures to improve responsiveness. These measures should include improving higher level hospital service, shortening waiting time, increasing the numbers of hospitals in rural regions, improving the medical environment, and expanding infrastructures to provide comfortable medical environments. The impact of such measures on responsiveness and the related factors will then need to be determined.

## Additional file

Additional file 1: Questionnaire. (DOCX 33 kb)
