# Supplementary material for: Healthcare system responsiveness in Jiangsu Province, China
Source: BMC Health Serv Res. 2017 Jan 13;17:31. doi: 10.1186/s12913-017-1980-2 (PMC5237227; doi:10.1186/s12913-017-1980-2)
Supplement: Additional file 1: — Questionnaire. (DOCX 33 kb) [file 12913_2017_1980_MOESM1_ESM.doc]

**Key Informant Survey on Responsiveness**

1、In the course of medical treatment and health education, doctors, nurses and other medical staff whether respect for you.

①never ②sometimes ③usually ④always

2、In the process of examination or treatment, your privacy (especially the body parts) is protected by medical staff?

①never ②sometimes ③usually ④always

3、Do you get a secret when you go to see a doctor? (You don't want anyone to know about your condition.

①never ②sometimes ③usually ④always

4、Did the medical staff ask for your opinion on the examination and treatment before and after the examination or treatment?

①never ②sometimes ③usually ④always

5、When you go to see a doctor, the doctor and nurse give you time to ask about your health and treatment issues?

①never ②sometimes ③usually ④always

6、Does the doctor explain the condition to you in a way that you understand and inform you of the conditions of treatment and inspection?

①never ②sometimes ③usually ④always

7、Does the medical staff provide you with information about treatment options for you to choose from?

①never ②sometimes ③usually ④always

8、Could you choose a doctor to see a doctor?

①never ②sometimes ③usually ④always

9、Do doctors allow you to choose another medical institution to see a doctor?

①never ②sometimes ③usually ④always

10、Is it convenient for you to get the health service when you need it?

①yes ②no

11、When needed, how long will it take you to get access to health care in the vicinity of your home?

①within one hour ②one hour ~ ③one and half of hour ~ two hour ④above two hour

12、How long you and your family usually have to wait to get related services in the health service institutions over the past year ?

①under five minute ②five minute~ ③ten minute~ ④thirty minute ~ ⑤ sixty minute and over

13、How long you and your family usually have to wait to get laboratory tests or results in the health service institutions over the past year?

①under ten minute ②ten minute~ ③thirty minute~ ④one hour ~ ⑤ two hour and over

14. Are you satisfied with the hospital's environment?

①satisfied ②moderate ③dissatisfied

15. Are you satisfied with the facilities and equipment of the hospital?

①satisfied ②moderate ③dissatisfied
